# Secondary coordination sphere accelerates hole transfer for enhanced hydrogen photogeneration from [FeFe]-hydrogenase mimic and CdSe QDs in water

**DOI:** 10.1038/srep29851

**Published:** 2016-07-15

**Authors:** Min Wen, Xu-Bing Li, Jing-Xin Jian, Xu-Zhe Wang, Hao-Lin Wu, Bin Chen, Chen-Ho Tung, Li-Zhu Wu

**Affiliations:** 1 Key Laboratory of Photochemical Conversion and Optoelectronic Materials, Technical Institute of Physics and Chemistry & University of Chinese Academy of Sciences, Chinese Academy of Sciences, Beijing 100190, P. R. China

## Abstract

Achieving highly efficient hydrogen (H_2_) evolution *via* artificial photosynthesis is a great ambition pursued by scientists in recent decades because H_2_ has high specific enthalpy of combustion and benign combustion product. [FeFe]-Hydrogenase ([FeFe]-H_2_ase) mimics have been demonstrated to be promising catalysts for H_2_ photoproduction. However, the efficient photocatalytic H_2_ generation system, consisting of PAA-*g*-Fe_2_S_2_, CdSe QDs and H_2_A, suffered from low stability, probably due to the hole accumulation induced photooxidation of CdSe QDs and the subsequent crash of [FeFe]-H_2_ase mimics. In this work, we take advantage of supramolecular interaction for the first time to construct the secondary coordination sphere of electron donors (HA^−^) to CdSe QDs. The generated secondary coordination sphere helps realize much faster hole removal with a ~30-fold increase, thus leading to higher stability and activity for H_2_ evolution. The unique photocatalytic H_2_ evolution system features a great increase of turnover number to 83600, which is the highest one obtained so far for photocatalytic H_2_ production by using [FeFe]-H_2_ase mimics as catalysts.

Nature has created [FeFe]-hydrogenase ([FeFe]-H_2_ase), a type of metalloenzyme in some specific bacteria and algaes, as a H_2_-forming catalyst with a high turnover rate (6000–9000 s^−1^ per catalytic site) at low over-potential^1^,^2^. The challenge is, however, to isolate the enzyme in large scale, and the isolated natural [FeFe]-H_2_ase would lose its activity once exposure to air^3^,^4^. To develop effective catalysts for H_2_ evolution, which is considered to be an extremely clean and renewable fuel to deal with energy crisis and environmental pollution^5^,^6^,^7^,^8^,^9^, scientists have devoted considerable efforts to simulating the active site of [FeFe]-H_2_ase^10^,^11^,^12^. Since the first attempt to fabricate artificial [FeFe]-H_2_ase based photosynthetic system^13^, [FeFe]-H_2_ase mimics have been demonstrated to be a category of cost-effective and efficient catalysts for H_2_ evolution under visible-light irradiation^14^,^15^,^16^,^17^,^18^. In particular, the efficiency of photocatalytic H_2_ evolution has been dramatically enhanced by combining [FeFe]-H_2_ase mimics with semiconductor quantum dots (QDs) in aqueous solution^14^,^19^,^20^, which benefits from both the intrinsic ability for proton reduction of [FeFe]-H_2_ase mimics and the advantage in light-absorbing and charge-separation of colloidal QDs^21^. In 2011, we used a water-soluble [FeFe]-H_2_ase mimic as catalyst, CdTe QDs as photosensitizer and ascorbic acid (H_2_A) as proton source and electron donor to construct an artificial photosynthetic system. Under the optimal condition, the turnover number (TON) of the system reached 505^22^. Whereafter, poly(acrylic acid)-based artificial [FeFe]-H_2_ase (PAA-*g*-Fe_2_S_2_) has been intergrated with CdSe QDs in H_2_A aqueous solution (pH 4.0) to achieve H_2_ production, improving TON value high over 20000 under visible-light irradiation^23^. Nevertheless, these [FeFe]-H_2_ase mimics and QDs based multi-component systems suffer from poor stability with gradual decomposion of both QDs and [FeFe]-H_2_ase mimics during irradiation, which is probably due to the hole transfer rate slower than the electron transfer rate^23^,^24^. We found, for example, that the electron transfer rate from CdSe QDs to the active site of PAA-*g*-Fe_2_S_2_ is three-order faster than the hole transfer rate from CdSe QDs to H_2_A^23^. In this regard, the cease of H_2_ evolution might be a result of accumulating holes in the valence band of CdSe QDs. One may question whether the stability and efficiency of photocatalytic H_2_ evolution system could be enhanced by means of facilitating hole removal.

In this contribution, we wish to report that the use of secondary coordination sphere greatly improves the activity and stability of the artificial [FeFe]-H_2_ase-based system, consisting of CdSe QDs, PAA-*g*-Fe_2_S_2_ and H_2_A in water (Fig. 1). Although PAA played a role in stabilizing CdSe QDs to some extent^23^, the protonation of carboxylic groups under acidic condition would reduce the association of PAA with CdSe QDs^25^. Further, the negatively charged carboxyl groups would prevent CdSe QDs from negatively charged HA^−^ in proximity. In this situation, we envision that addition of polyethyleneimine (PEI) would be helpful for the intimate interaction of CdSe QDs with PAA-*g*-Fe_2_S_2_ and H_2_A because PEI has the ability to protect cadmium chalcogenide nanocrystals from aggregation in an extremely wide pH range^26^,^27^. Unlike the most state-of-the-art approaches^28^,^29^,^30^,^31^,^32^,^33^, we take advantage of supramolecular interaction to increase the contact between CdSe QDs and electron donors (HA^−^). The positive charge of PEI under acidic condition^34^ not only endows CdSe QDs a capacity to associate with PAA-*g*-Fe_2_S_2_ intimately, but also enables HA^−^ to contact closely with CdSe QDs than that in the absence of PEI. Indeed, the as-generated secondary coordination sphere helps with acceleration of hole transfer to a ~30-fold increase, while the electron transfer to PAA-*g*-Fe_2_S_2_ remains unchanged. As a result, the designed system can catalyze proton reduction under visible-light irradiation for 28 h, giving rise to a TON value high up to 83600. This is, to the best of our knowledge, the highest value known to date based on [FeFe]-H_2_ase mimics for H_2_ photogeneration.

## Results

### Photocatalytic H_2_ generation

The H_2_ evolution experiments were carried out in argon saturated aqueous solution of CdSe QDs, PAA-*g*-Fe_2_S_2_ and H_2_A at pH 4.1 under visible-light irradiation. PAA-*g*-Fe_2_S_2_ and CdSe QDs were prepared according to the reported procedures^23^. The characterizations of PAA-*g*-Fe_2_S_2_ and CdSe QDs were described in Supplementary Information (Figs S1 and S2). As shown in Fig. 2, the photocatalytic system, containing CdSe QDs, PAA-*g*-Fe_2_S_2_ and H_2_A at pH 4.1, ceased to evolve H_2_ in 4 h, but a significant enhancement of H_2_ evolution was achieved in the presence of 0.12 g·L^−1^ PEI under the same condition (Supplementary Fig. S3). Increasing the concentration of PEI to 0.46 g·L^−1^ improved the rate of H_2_ production (Supplementary Fig. S4) till higher concentration to 1.84 g·L^−1^. The excessive amount of PEI probably retarded the interaction between CdSe QDs and H_2_A and/or PAA-*g*-Fe_2_S_2_ because the average hydrodynamic diameter of CdSe QDs remained unchanged (Supplementary Fig. S5). Note that the rate of H_2_ evolution was positively proportional to the concentration of H_2_A in the range of 0 to 0.1 mol·L^−1^ (Supplementary Fig. S6), and negligible H_2_ was obtained in the absence of H_2_A (0 mol·L^−1^), PEI and PAA themselves were not possible to serve as electron donors in the designed system. Control experiment, performed under the same concentrations of CdSe QDs, H_2_A, PAA and PEI, evolved much less H_2_, which confirmed the role of Fe_2_S_2_ for proton reduction (Supplementary Fig. S7). To our delight, the rate of H_2_ production was linear in 10 h and the lifetime of H_2_ evolution could be prolonged to 28 h, yielding an unprecedented TON value to 83600 (Fig. 2).

### Influence of PEI to the stability of CdSe QDs

The presence of PEI could improve the efficiency and stability of the CdSe QDs and PAA-*g*-Fe_2_S_2_ system for photocatalytic H_2_ evolution. To verify the function of PEI, dynamic light scattering (DLS) and high-resolution transmission electron microscope (HRTEM) were employed. As shown in Fig. 3a, CdSe QDs dispersed well without any mutual aggregation under neutral condition. Upon introduction of 0.1 mol·L^−1^ H_2_A (pH 4.1) to the solution, the average hydrodynamic size of CdSe QDs increased dramatically to ~1400 nm and severe aggregation of CdSe QDs was seen directly from the corresponding TEM image (Fig. 3b), a result of dissociation of surface ligands, mercaptopropionic acid (MPA), from CdSe QDs at acidic condition^35^. When a certain amount of PAA-*g*-Fe_2_S_2_ (0.25 g·L^−1^) was added into the solution, the hydrodynamic size of CdSe QDs decreased from ~1400 nm to ~192 nm. The better dispersion of CdSe QDs in the presence of PAA-*g*-Fe_2_S_2_ was well reflected by TEM image (Fig. 3c), which provided evidence on the stabilization of PAA to CdSe QDs since there are no chemical groups in Fe_2_S_2_ cluster can stabilize CdSe QDs. The better dispersity of CdSe QDs caused by the coordination of PAA on the surface could also be confirmed by the enhanced emission intensity and blue-shift of band edge emission (Supplementary Fig. S8). More strikingly, when PEI (0.46 g·L^−1^) was simultaneously introduced into the solution of CdSe QDs and PAA-*g*-Fe_2_S_2_, the average size distribution of CdSe QDs decreased to merely ~17 nm, which could be comparable to that in neutral water (Fig. 3d). Also, high-resolution TEM indicated the excellent dispersity of CdSe QDs in the co-presence of PAA and PEI (Fig. 3d). Clearly, the coordination between amino groups on PEI^36^,^37^ with Cd^2+^ is so strong even under acidic condition that the presence of PEI allows an excellent dispersion of CdSe QDs at pH 4.1. The solution of the *in situ* generated PEI and PAA co-stabilized CdSe QDs was found stable for at least 7 days (Supplementary Fig. S9). The better stability of CdSe QDs in the presence of PEI ensured better interaction with PAA-*g*-Fe_2_S_2_, which in turn improved the efficiency and stability of this photocatalytic H_2_ production system.

Consistent with the observation of HRTEM and DLS studies, an apparent blue shift of the CdSe QDs emission in the presence of PEI (Table 1 and Supplementary Fig. S8) suggested that PEI could stabilize CdSe QDs and prevent the formation of large aggregates^27^. Furthermore, with the addition of PEI (0.46 g·L^−1^) into the solution of CdSe QDs and PAA, the emission decay was apparently facilitated from 14.7 ns to 9.6 ns (Supplementary Fig. S10). With reference to the reports in literature^38^,^39^,^40^, the shortened lifetime could be attributed to the delocalization of photo-generated excitons, especially holes, to the surface layer of PEI, which would further benefit hole depletion of CdSe QDs by relaying holes to sacrificial reagents.

### Influence of PEI to the electron transfer rate of CdSe QDs

The reductive potential of PAA-*g*-Fe_2_S_2_ was determined to be −0.43 V (*vs* NHE), demonstrating an exothermic electron transfer from excited CdSe QDs to PAA-*g*-Fe_2_S_2_ because the conduction band potential of CdSe QDs is more negative than −0.43 V (*vs* NHE)^23^. Spectroelectrochemical and time-resolved absorption spectra were then used to study the electron transfer and hole transfer process in the designed system^41^,^42^,^43^,^44^. Either in the absence or presence of PEI, electrochemical reduction of PAA-*g*-Fe_2_S_2_ led to the formation of a new absorption peak around 400 nm, which could be assigned to Fe^I^Fe^0^ species (Supplementary Fig. S11)^45^,^46^,^47^. Under the same condition, electrochemical reduction of PAA and PEI couldn’t result in the formation of similar signals (Supplementary Fig. S12). The results suggested that the introduction of PEI would not influence the active site of PAA-*g*-Fe_2_S_2_ very much. Time-resolved transient absorption spectra were measured to further evaluate the electron transfer from CdSe QDs to PAA-*g*-Fe_2_S_2_. As PAA-*g*-Fe_2_S_2_ was added into the aqueous solution of CdSe QDs, the bleaching recovery of CdSe QDs was apparently facilitated from 36.9 ns to 11.5 ns, a result of electron transfer from excited CdSe QDs to the active site of PAA-*g*-Fe_2_S_2_ (Fig. 4, Supplementary Table S1)^23^,^41^. The rate of electron transfer (*k*_e_) was determined^41^ to be 3.4 × 10^11^ M^−1^·s^−1^ and 4.5 × 10^11^ M^−1^·s^−1^ in the absence and presence of PEI (0.46 g·L^−1^), respectively (Table 1, see details in Supplementary Information). Obviously, the introduction of PEI could hardly change electron transfer from CdSe QDs to PAA-*g*-Fe_2_S_2_ for proton reduction.

### Influence of PEI to the hole transfer rate of CdSe QDs

The hole transfer kinetics from CdSe QDs to HA^−^ was carefully examined^42^,^43^. Progressive addition of ascorbate sodium (NaHA) to aqueous solutions, *i.e.* CdSe QDs and PAA; CdSe QDs, PAA and PEI, at pH 4.1, respectively, resulted in emission quenching of CdSe QDs to varied extent. Combining with the emission decay with and without PEI (Supplementary Fig. S10), the rate of hole transfer (*k*_h_) from CdSe QDs to HA^−^ was determined as 1.5 × 10^10^ M^−1^·s^−1^ and 4.6 × 10^8^ M^−1^·s^−1^ (Table 1, Fig. 5, Supplementary Figs S13 and S14, see details in Supplementary Information). Surprisingly, a ~30-fold enhancement of hole transfer was obtained with the addition of PEI. The presence of PEI facilitated the hole-extraction pathway of CdSe QDs by HA^−^. Considering the coordination of amino groups of PEI on the surface of CdSe QDs, the superficial electric charge of CdSe QDs would be altered. Indeed, the zeta potentials (ζ) of CdSe QDs in H_2_A (0.1 mol·L^−1^) solution, and in H_2_A (0.1 mol·L^−1^) and PAA (0.25 g·L^−1^) solution were measured to be −4.7 mV and −8.7 mV, respectively (Table 1, Supplementary Table S2). After adding PEI (0.46 g·L^−1^) into the solution, the ζ value changed to +25.7 mV, indicating the extremely strong positive charge of PEI modified CdSe QDs. On the one hand, the strongly positive charge ensured the excellent stability of CdSe QDs in water due to the strong electrostatic repulsion, *vide ante* (Supplementary Fig. S9). On the other hand, the inversed charge of CdSe QDs surface provided a driving force to interact with negatively charged HA^−^ by electrostatic interaction. The as-generated secondary coordination sphere strengthened the association of HA^−^ with CdSe QDs, which in turn accelerated the hole transfer from CdSe QDs to HA^− ^48.

The accelerated hole transfer process is well-manifested by the model of electrical double layer (EDL) to estimate the concentration of adsorbed counterion (see details in Supplementary Information)^49^. For PAA and PEI/PAA stabilized CdSe QDs, the surface adsorbed HA^−^ ions were determined as 0.07 mol·L^−1^ and 0.27 mol·L^−1^, respectively. The secondary coordination sphere of HA^−^ to CdSe QDs, simply by addition of PEI, contributed a ~30-fold increase of hole transfer from CdSe QDs to HA^−^ and a significant decrease of *k*_e_/*k*_h_ ratio from ~740 to ~30, which benefited the balance of excitons consumption and protected CdSe QDs from photo-oxidation during visible-light irradiation. As a result, the CdSe QDs and PAA-*g*-Fe_2_S_2_ system that would decompose in 4 h irradiation showed a remarkably enhanced activity and stability for photocatalytic H_2_ evolution.

## Discussion

**Figure 1:**
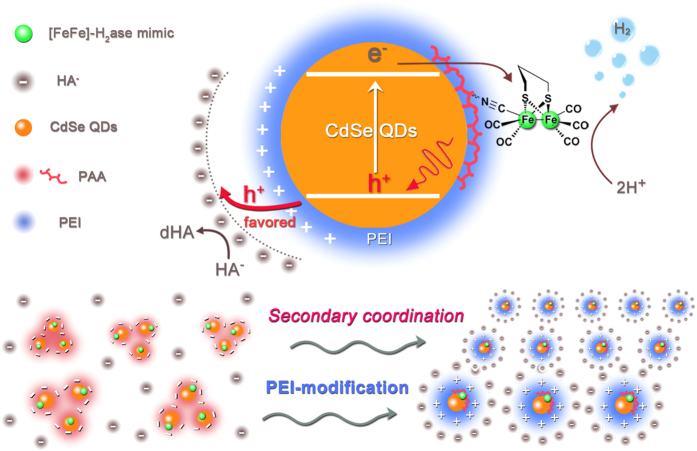
Schematic illustration of the secondary coordination sphere *via* PEI-modification. The dispersion of the system without (left panel) and with PEI (right panel) and the secondary coordination sphere enhanced process of photocatalytic H_2_ evolution.

**Figure 2:**
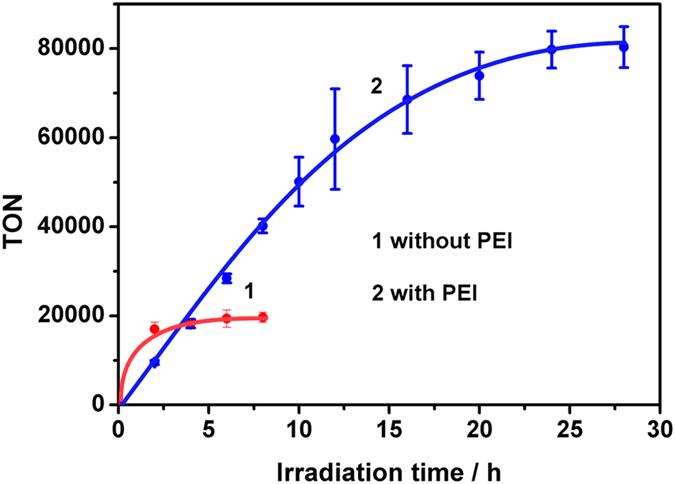
Photocatalytic H_2_ production in the absence (line 1) and presence (line 2) of PEI. Concentrations: CdSe QDs (5.8 × 10^−6^ mol·L^−1^), PAA-*g*-Fe_2_S_2_ (0.25 g·L^−1^), H_2_A (0.1 mol·L^−1^), PEI (0.46 g·L^−1^), pH 4.1.

**Figure 3:**
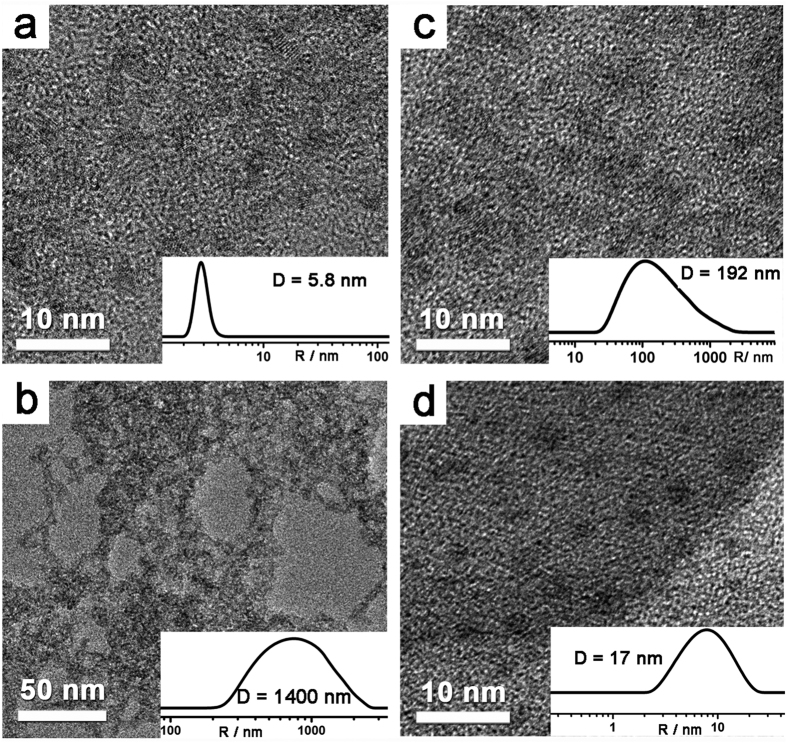
TEM images and corresponding DLS profiles (the inset panels) of CdSe QDs in water under different conditions. (**a**) In neutral water, pH 6.9; (**b**) in the presence of H_2_A, pH 4.1; (**c**) in the presence of H_2_A and PAA-*g*-Fe_2_S_2_, pH 4.1; (**d**) in the presence of H_2_A, PAA-*g*-Fe_2_S_2_ and PEI, pH 4.1. Concentrations: CdSe QDs (5.8 × 10^−6^ mol·L^−1^), H_2_A (0.1 mol·L^−1^), PAA-*g*-Fe_2_S_2_ (0.25 g·L^−1^), PEI (0.46 g·L^−1^).

**Figure 4:**
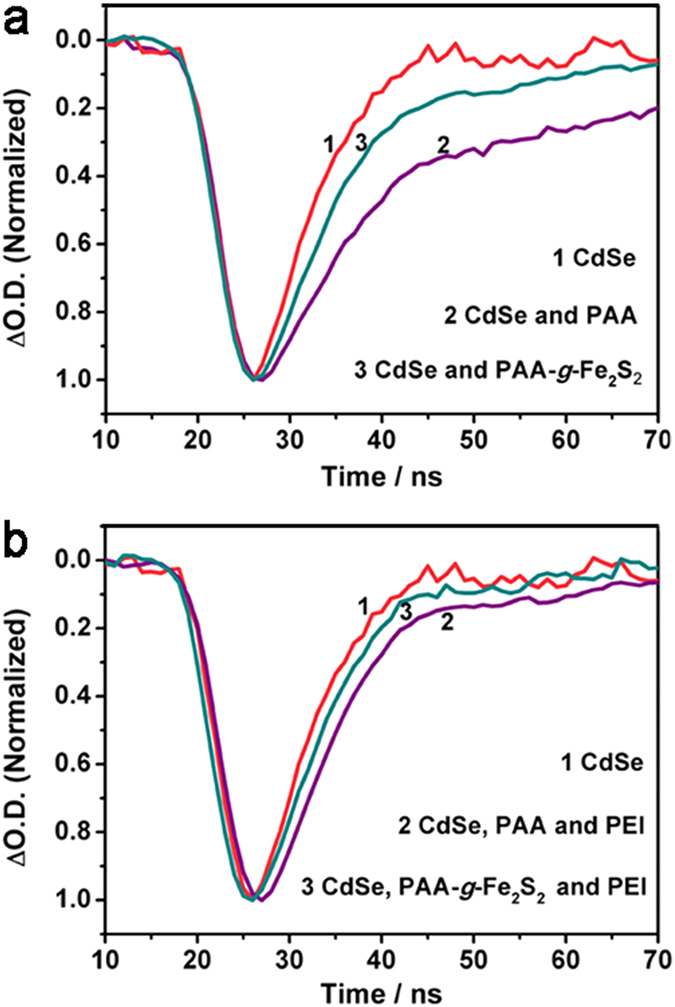
Transient absorption measurements. (**a**) The recovery kinetics of CdSe QDs at 430 nm in water at pH 4.1 (line 1), in the presence of PAA (line 2) and PAA-*g-*Fe_*2*_S_*2*_ (line 3); (**b**) the recovery kinetics of CdSe QDs at 430 nm in water at pH 4.1 (line 1), in the presence of PAA and PEI (line 2), and PAA-*g-*Fe_*2*_S_*2*_ and PEI (line 3). Concentrations: CdSe QDs (1.6 × 10^−5^ mol·L^−1^), PAA (0.25 g·L^−1^), PAA-*g*-Fe_2_S_2_ (0.25 g·L^−1^), PEI (0.46 g·L^−1^), pH 4.1.

**Figure 5:**
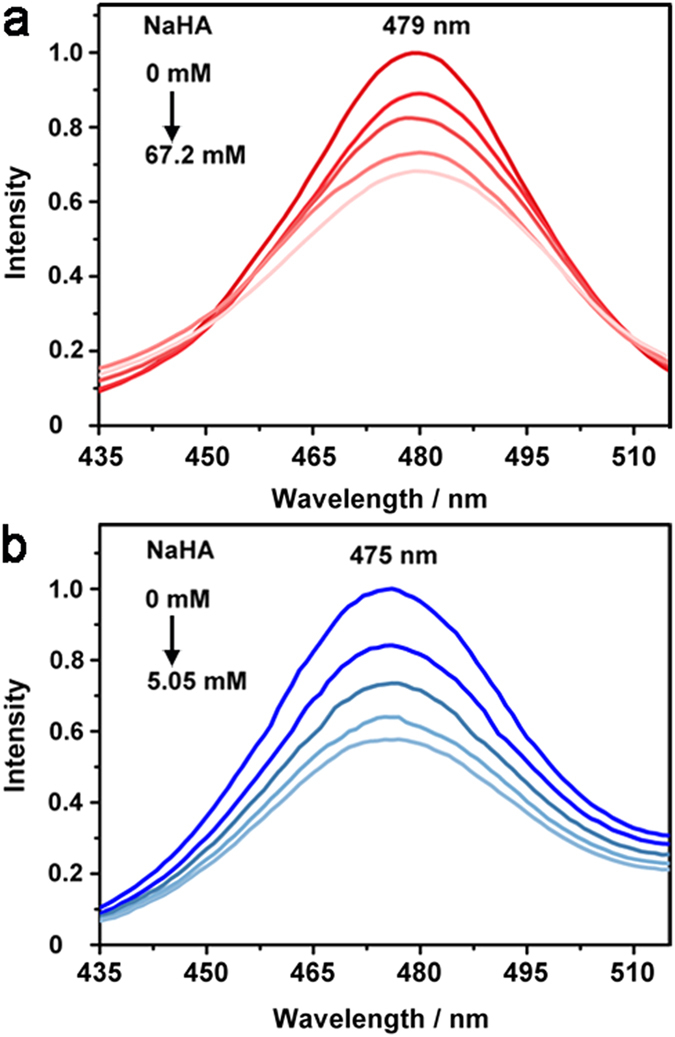
Hole depletion of CdSe QDs. The emission quenching of CdSe QDs with gradual addition of NaHA in the presence of PAA (**a**) and in the presence of PAA and PEI (**b**). Concentrations: CdSe QDs (1.6 × 10^−5^ mol·L^−1^), PAA (0.25 g·L^−1^), PAA-*g*-Fe_2_S_2_ (0.25 g·L^−1^), PEI (0.46 g·L^−1^), pH 4.1.

On the basis of above results, we speculated that the secondary coordination sphere is crucial for the enhancement of H_2_ photogeneration from [FeFe]-H_2_ase mimic and CdSe QDs in water. Upon photoexcitation, CdSe QDs induces electrons promoting to the conduction band and leaving holes in the valence band to form separated electron/hole pairs. Photoexcited electron in the conduction band of CdSe QDs transfers to the Fe_2_S_2_ core of PAA-*g*-Fe_2_S_2_ and generates a one-electron reduced Fe^I^Fe^0^ species. This active species further reacts with a proton in catalytic cycle to produce H_2_. At the same time, the leaving hole in the valence band of CdSe QDs is captured by electron donor HA^−^ to regenerate CdSe QDs. Because two electrons are required to produce each molecular H_2_, the regeneration of both CdSe QDs and Fe_2_S_2_ active site of [FeFe]-H_2_ase mimic is imperative. So the better balance of electron transfer and hole transfer of CdSe QDs to PAA-*g*-Fe_2_S_2_ and HA^−^ is, the higher efficiency of H_2_ evolution would be. In this typical PAA-*g*-Fe_2_S_2_-based system, the *in situ* PEI-modification can greatly improve the stability of CdSe QDs to a well-dispersed colloidal solution under acidic condition (Fig. 1), which ensures smooth electron transfer from CdSe QDs to PAA-*g*-Fe_2_S_2_. More importantly, the *in situ* PEI modification induces the secondary coordination of negatively charged sacrificial reagents (HA^−^) to the positively charged surface of the modified CdSe QDs, which contributes a 30-fold enhancement of hole transfer rate. Benefitting from these advantages, the photocorrosion of CdSe QDs is avoided to a great extent by facilitating hole extraction, and thus greatly enhancing the efficiency and stability of CdSe QDs and PAA-*g*-Fe_2_S_2_ system for photocatalytic H_2_ evolution.

In summary, we have demonstrated for the first time that secondary coordination sphere enables to facilitate hole transfer for efficient H_2_ photogeneration. In terms of supramolecular interaction, the performance of photocatalytic system, *i.e.* CdSe QDs, PAA-*g*-Fe_2_S_2_, and H_2_A at pH 4.1, has been improved by PEI to 83600 turnovers, which is the highest value known to date for photocatalytic H_2_ evolution by using [FeFe]-H_2_ase mimics as catalysts. Studies on steady-state and time-resolved spectroscopy reveal that the presence of PEI greatly enhances the rate of hole transfer up to ~30-fold as compared with the same system without PEI. As a result, the ratio (*k*_e_/*k*_h_) of electron transfer and hole transfer from CdSe QDs to PAA-*g*-Fe_2_S_2_ and HA^−^ decreases from 740 to 30, which is advantageous to the balance of excitons generated by photoexcited CdSe QDs to synergistically improve the efficiency of H_2_ evolution. Our results imply that environment surrounding each component, for example, CdSe QDs photosensitizer, Fe_2_S_2_ catalyst and H_2_A sacrifical electron donor and proton source in this case, might cause significant activity difference of photocatalytic system. The crucial role of PEI suggests that to create efficient H_2_ evolution systems based on [FeFe]-H_2_ase mimic, one would need to mimic not only the active site structure of [FeFe]-H_2_ase but also the kinetic balance for electron transfer and hole transfer in a real H_2_ evolution system. It is anticipated that the use of secondary coordination sphere would be a promising alternative to enhance the stability and efficiency of artifical [FeFe]-H_2_ase-based system for H_2_ evolution, which is reminiscent of natural [FeFe]-H_2_ase buried in protein matrix.

## Methods

### H_2_ evolution experiments

A typical procedure for H_2_ production was described as follows. Certain amounts of PAA-*g*-Fe_2_S_2_, MPA-CdSe QDs, H_2_A and PEI were dissolved in ultrapure water respectively, to make a solution at certain concentration. Then, certain volumes of the solutions for PAA-*g*-Fe_2_S_2_, MPA-CdSe QDs, H_2_A and PEI were taken to mix in a Schlenk tube. The pH value of the solutions were determined by a pH meter and adjusted by aqueous NaOH or HCl solution. The total volume of the mixed solution was diluted with ultrapure water to 5 mL. Thereafter, the sample was saturated with argon gas and 1000 μL CH_4_ was injected as the internal standard for quantitative GC-TCD analysis. The light source was a blue LED lamp (3 W, λ = 450 nm) equipped with an agitator and a cooling apparatus. After a certain period of irradiation time, 500 μL mixed gas was taken from the sample tube and injected into the GC for analysis. The response factor for H_2_ was 8.47 and the response factor for CH_4_ was 2.09 under the experimental condition, which were established by calibration with known amounts of H_2_ and CH_4_, and determined before and after a series of measurements.

### Synthesis of MPA-CdSe QDs

MPA-CdSe QDs were synthesized according to the literature with slight modification^50^. Briefly, selenium powder (40 mg) was added into Na_2_SO_3_ aqueous solution (100 mL, 1.5 mmol). The mixture was refluxed at 130 degree until the solid powder of selenium disappeared and then colourless transparent Na_2_SeSO_3_ solution was obtained. The Na_2_SeSO_3_ solution (10 mL) was extracted and injected into the argon saturated CdCl_2_ solution (46 mg CdCl_2_·2.5H_2_O in 190 mL ultrapure water) at pH 11 in the presence of 3-mercaptopropionic acid (26 uL) as stabilizing agent. The resulting mixture was refluxed at 120 degree to control the growth of CdSe QDs. The size of QDs was monitored by UV-vis absorption spectrum during refluxing.

### Synthesis of PAA-*g*-Fe_2_S_2_

The water soluble catalyst PAA-*g*-Fe_2_S_2_ was synthesized according to our previous procedure^23^. The color of pure PAA is white and the color of pure Fe_2_S_2_ active site is red. After modification, the color of PAA changed from white to light red, indicating the successful anchor of Fe_2_S_2_ on the chain of PAA. ^1^H-NMR, UV/Vis spectrum and FTIR spectroscopy further confirmed the modification of the Fe_2_S_2_ active site onto the chain of PAA. The grafting amount of Fe_2_S_2_ was determined as 2.8 × 10^−6^ mol·g^−1^ for PAA-*g*-Fe_2_S_2_ by inductively coupled plasma-atomic emission spectrometry (ICP-AES) based on the relative content of Fe in samples. Spectroelectrochemical and time-resolved absorption spectra experiments employed PAA-*g*-Fe_2_S_2_ with larger grafting amount of Fe_2_S_2_ site (7.1 × 10^−4^ mol·g^−1^) as electron capture to obtain more obvious signals.

Details of the instruments, chemicals and the computing methods used in this work are given in the Supplementary Information.

## Additional Information

**How to cite this article**: Wen, M. *et al*. Secondary coordination sphere accelerates hole transfer for enhanced hydrogen photogeneration from [FeFe]-hydrogenase mimic and CdSe QDs in water. *Sci. Rep.* **6**, 29851; doi: 10.1038/srep29851 (2016).

## Supplementary Material

Supplementary Information

## Figures and Tables

**Table 1:** Properties of systems for H_2_ evolution in the presence and absence of PEI.

**Systems**	**λ**_**e**_**/nm**	**τ**_**e**_**/ns**	***k***_**e**_**/M**^**−1**^**·s**^**−1**^	***k***_**h**_**/M**^**−1**^**·s**^**−1**^	**ζ/mV**	**TON**	***t***_***lifetime***_**/h**
without PEI	479	14.7	3.4 × 10^11^	4.6 × 10^8^	−8.7	20000	4
with PEI	475	9.6	4.5 × 10^11^	1.5 × 10^10^	+25.7	83600	28

(λ_e_) Emission peak of CdSe QDs; (τ_e_) Emission lifetime of CdSe QDs; (*k*_e_) The rate of electron transfer from CdSe QDs to PAA-*g*-Fe_2_S_2_; (*k*_h_) The rate of hole transfer from CdSe QDs to H_2_A; (ζ) Zeta potential; (TON) Turnover number of the systems; (*t*_*lifetime*_) The time that the systems could catalyze proton reduction.
