# Supplementary material for: Secondary coordination sphere accelerates hole transfer for enhanced hydrogen photogeneration from [FeFe]-hydrogenase mimic and CdSe QDs in water
Source: Sci Rep. 2016 Jul 15;6:29851. doi: 10.1038/srep29851 (PMC4945928; doi:10.1038/srep29851)
Supplement: Supplementary Information [file srep29851-s1.doc]

**Supplementary Information**

**Secondary coordination sphere accelerates hole transfer for enhanced hydrogen photogeneration from** **[FeFe]-hydrogenase mimic and CdSe QDs in water**

Min Wen, Xu-Bing Li*, Jing-Xin Jian, Xu-Zhe Wang, Hao-Lin Wu, Bin Chen, Chen-Ho Tung and Li-Zhu Wu*

Key Laboratory of Photochemical Conversion and Optoelectronic Materials, Technical Institute of Physics and Chemistry & University of Chinese Academy of Sciences, the Chinese Academy of Sciences, Beijing 100190, P. R. China

*To whom correspondence should be addressed. *E-mail:* [lixubing@mail.ipc.ac.cn](mailto:lixubing@mail.ipc.ac.cn), [lzwu@mail.ipc.ac.cn](mailto:lzwu@mail.ipc.ac.cn)

*Telephone:* (+86) 10-8254-3580*, Fax:* (+86) 10-8254-3580

**Table of Contents**

1. Instruments

2. Chemicals

3. The size, extinction coefficient (*ε*) and concentration (*c*) of CdSe QDs

4. Average emission lifetime

5. Calculation of electron transfer rate

6. Calculation of hole transfer rate

7. Calculation of the adsorbed counterion

8. Characterization of PAA-*g*-Fe_2_S_2_

9. UV/Vis absorption spectrum of CdSe QDs

10. H_2_ production in the absence and presence of PEI

11. H_2_ production with different amounts of PEI

12. DLS results of CdSe QDs with different amounts of PEI

13. H_2_ production with different amounts of H_2_A

14. H_2_ production in the absence and presence of Fe_2_S_2_ active site

15. Emission spectra of CdSe QDs under different conditions

16. Photographs of PEI-modified CdSe QDs

17. Emission decays of CdSe QDs with and without PEI

18. Spectroelectrochemical absorption spectra of Fe^I^Fe^0^ species

19. Spectroelectrochemical reduction of PAA and PEI

20. Stern-Volmer plot describing emission quenching of CdSe QDs

21. Stern-Volmer plot describing emission quenching of CdSe QDs with PEI

22. Reference

1. Instruments

UV/Vis absorption spectra were measured on a Shimadzu UV-1601PC spectrophotometer in a quartz cell with an optical length of 1 cm. Emission spectra were recorded on a Hitachi 4500 fluorescence spectrophotometer. Luminescence decay measurements were performed with a FLS-920 (Edinburgh Instruments Ltd., UK) apparatus with 405 nm laser excitation (pulse width *ca.* 100 ps). Time-resolved decay measurements of the bleaching of CdSe QDs were provided by using an OPO laser at 410 nm. The prober was a xenon lamp on the Edinburgh LP-920 apparatus from analytical instruments. The amount of Fe_2_S_2_ active site in PAA-*g*-Fe_2_S_2_ was determined by ICP-AES analysis (Varian 710-ES). Dynamic light scattering (DLS) was recorded on a Dybapro NanoStar (Wyatt, USA) scattering apparatus. High-resolution transmission electron microscopy (TEM) was performed on a JEM 2100F (Japan) electron microscope operated at an accelerating voltage of 200 kV. ^1^H-NMR spectrum was recorded on a Bruker 400 FT-NMR spectrometer and chemical shift is relative to tetramethylsilane. Fourier transform infra-red (FTIR) spectroscopy spectra were taken on Excalibur 3100 system (Varian, USA). All pH measurements were made with a Model pHS-3C meter (Mettler Toledo FE20, China). Gas chromatography (GC) was performed on a TIANMEI 7890-II using argon as the carrier gas with a molecular sieve column (5 Å; 2 m × 2 mm) and a thermal conductivity detector. The zeta potentials were measured using a Malvern Zetasizer 3000HS apparatus. Spectroelectrochemical experiments were performed in a quartz cell. Glass carbon electrode was used as the working electrode and platinum wire electrode and Ag/AgCl reference electrode were served as the counter and reference electrodes, respectively. The electrolyte solution was purged with argon for 30 min before the absorption spectra were recorded on a Shimadzu UV-1601PC spectrometer. Spectroelectrochemical absorption spectra were recorded along with time of electrochemical reduction of PAA-*g*-Fe_2_S_2_ at constant voltages, the baseline of which referred to the absorption of PAA-*g*-Fe_2_S_2_ or PAA-*g*-Fe_2_S_2_ and PEI before reduction under the constant voltage.

**2. Chemicals**

3-Mercaptopropionic acid (99%), CdCl_2_·5/2H_2_O (99%), Na_2_SO_3_ (99%) and Ascorbic acid (99%) were purchased from Alfa aesar. Selenium powder (about 200 mesh), Polyethyleneimine (M.W. 25000, branched) and Polyacrylic acid (M.W. 1800, linear) were purchased from Sigma-Aldrich. Other chemicals are of analytical grade and used without further purification unless otherwise noted. Ultrapure water with 18.2 MΩ cm (Mettler Toledo, FE20, China) was used thorough.

**3. The size, extinction coefficient (*ε*) and concentration (*c*) of CdSe QDs**

According to the equations reported by Peng and co-workers^1^, the diameter (*D*), extinction coefficient (*ε*) and concentration (*c*) of the CdSe QDs can be determined by using following equations:

*D* = (1.6122 × 10^-9^)*λ*^4^ - (2.6575 × 10^-6^)*λ*^3^ + (1.6242 × 10^-3^)*λ*^2^ - 0.4277*λ* + 41.57 (S1)

*ε* = 5857(*D*)^2.65^ (S2)

*A* = *εcL* (S3)

Here, *D* (nm) is the diameter or size of a given nanocrystal sample. *λ* is the wavelength of the first excitonic absorption peak of the corresponding sample. *ε* is the extinction coefficient of the corresponding sample. *A* is the absorbance of the sample, *L* (1 cm) is the length of cuvette in the direction of irradiation and *c* is the concentration of the corresponding sample. In our experiments, the diameter (*D*) of the MPA-CdSe QDs was determined as 1.9 nm and the extinction coefficient *ε* was 2.8 × 10^4^ (L·mol^-1^·cm^-1^) according to equation S1 and S2, respectively. The concentration of the obtained MPA-CdSe QDs aqueous solution was determined as 2.4 × 10^-5^ (mol·L^-1^) by using equation S3.

**4. Average emission lifetime**

The emission decays and characteristic bleaching decays of CdSe QDs in the absence and presence of PEI were studied and the decay traces for the samples were well fitted with triplet-exponential function *Y(t)* based on nonlinear least-squares, using the following expression^2-3^.

*Y(t)* = *B_1_*exp(-*t*/τ_1_) + *B_2_*exp(-*t*/τ_2_) + *B_3_*exp(-*t*/τ_3_)

where *B_1_, B_2_, B_3_* are fractional contributions of time-resolved emission decay lifetimes τ_1_, τ_2_, τ_3_.

The average lifetime τ can be determined by using equation S4:

<τ> = (*B_1_*τ_1_^2^ + *B_2_*τ_2_^2^ + *B_3_*τ_3_^2^)/(*B_1_*τ_1_ + *B_2_*τ_2_ + *B_3_*τ_3_) (S4)

**5. Calculation of electron transfer rate**

| **Table S1**: The decay rate of the bleaching of CdSe QDs at 430 nm in different systems. | | | | | |
| --- | --- | --- | --- | --- | --- |
| samples | CdSe QDs | CdSe QDs +  PAA | CdSe QDs + PAA-*g*-Fe_2_S_2_ | CdSe QDs + PAA+PEI | CdSe QDs + PAA-*g*-Fe_2_S_2_ + PEI |
| τ / ns | 3.6 | 36.9 | 11.5 | 8.5 | 5.1 |

According to the equation reported by Kamat^4-5^, the rate of electron transfer from CdSe QDs to PAA-*g*-Fe_2_S_2_ can be calculated by using equation S5:

*k*_e_·*c* = 1/τ - 1/τ_0_ (S5)

Here, *k*_e_ is the electron transfer rate; *c* is the concentration of the active site of PAA-*g*-Fe_2_S_2_; τ is the decay rate of the characteristic bleaching of CdSe QDs in the presence of PAA-*g*-Fe_2_S_2_; τ_0_ is the decay rate of the characteristic bleaching of CdSe QDs when PAA-*g*-Fe_2_S_2_ was replaced by PAA.

**6. Calculation of hole transfer rate**

The rate of hole transfer from CdSe QDs to electron donors can be calculated by Stern-Volmer equation^6-7^.

*k*_h_·τ_e0_·*c*_D_ = I_0_/I_p_ - 1 (S6)

Here, I_0_ is the emission intensity of CdSe QDs before addition of electron donors; I_p_ is the emission intensity of CdSe QDs when a certain amount of electron donors was added to the solution and *c*_D_ is the corresponding concentration of the electron donors; τ_e0_ is the average emission lifetime of CdSe QDs before the addition of electron donors; *k*_h_ is the rate of hole transfer.

**7. Calculation of the adsorbed counterion**

According to the model of electrical double layer (EDL)^8^, the concentration of adsorbed counterion could be calculated by equation S7:

*C_s_* = *C_B_* exp(*e * Ψ / κT*) (S7)

*C_s_* is the concentration of the adsorbed HA^-^, *C_B_* is the bulk concentration of HA^-^, *e* is the elementary charge, *κ* is the Boltzmann constant, *T* is the absolute temperature, and *Ψ* is the electric potential, which is equal to ζ here.

| **Table S2**: The Zeta potentials (ζ) of CdSe QDs in different systems at pH 4.1. | | | |
| --- | --- | --- | --- |
| systems | CdSe QDs | CdSe QDs +  PAA-*g*-Fe_2_S_2_ | CdSe QDs +  PAA-*g*-Fe_2_S_2_ + PEI |
| ζ / mV | -4.7 | -8.7 | +25.7 |

**8. Characterization of PAA-*g*-Fe_2_S_2_**


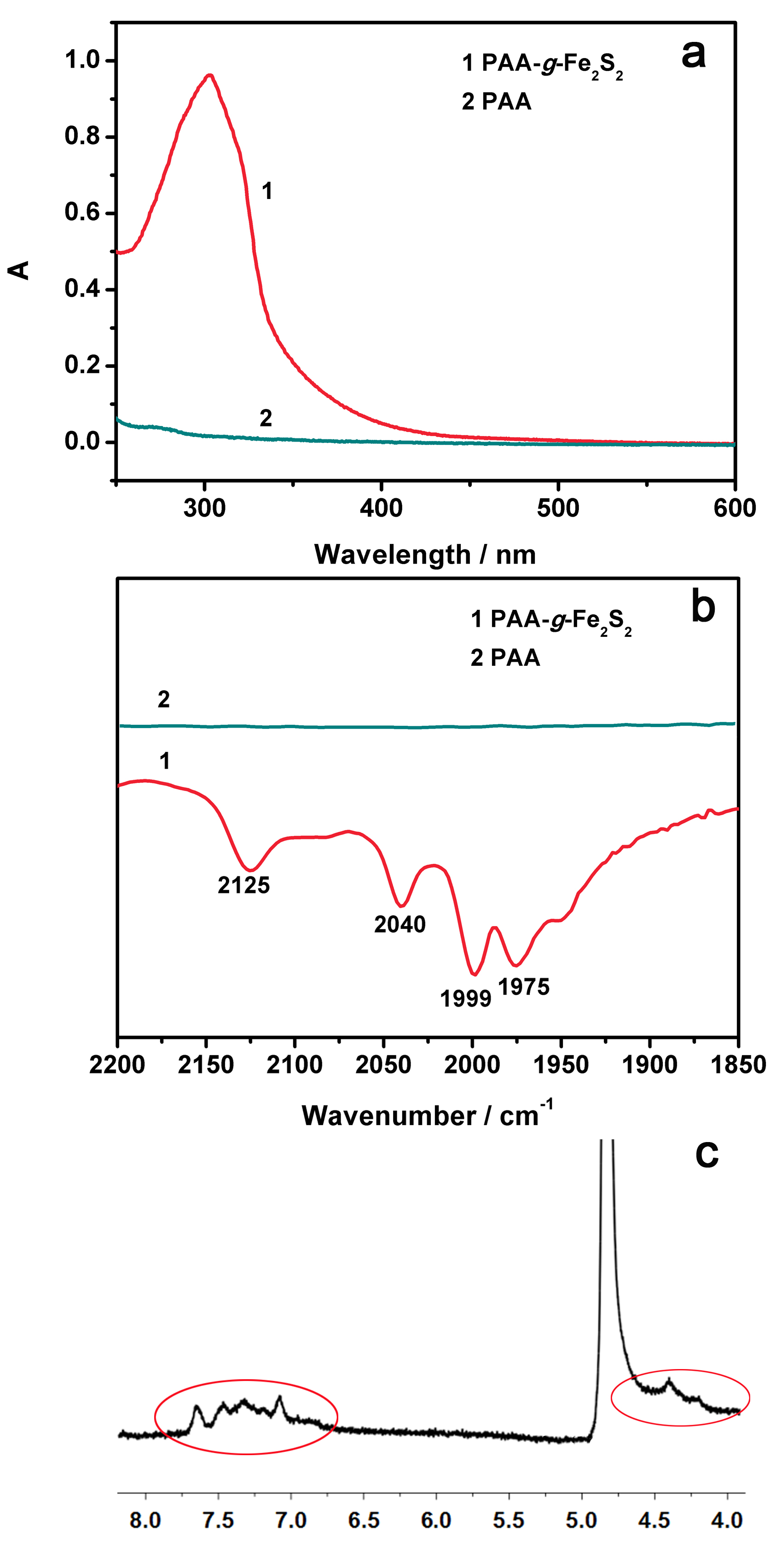


**Figure S1.** UV/Vis absorption spectrum (a), FTIR spectrum (b) and ^1^H-NMR (c) of PAA-*g*-Fe_2_S_2_. The concentration of sample for UV-Vis absorption spectrum was 0.13 g·L^-1^ in water and the ^1^H-NMR measurement was examined in D_2_O.

**9. UV-Vis absorption spectrum of CdSe QDs**


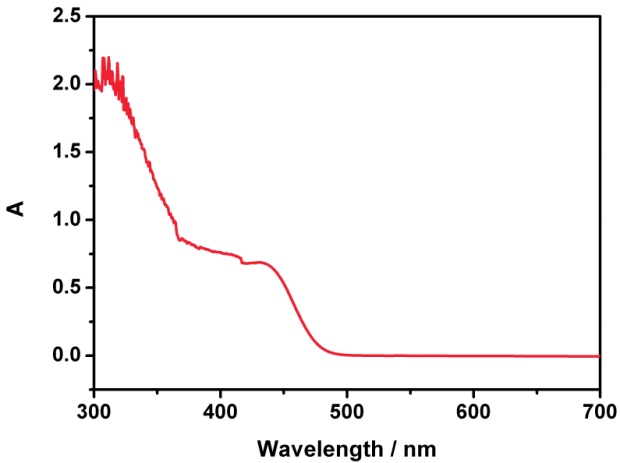


**Figure S2.** UV/Vis absorption spectrum of CdSe QDs (2.4 × 10^-5^ mol·L^-1^) in aqueous solution.

**10. H_2_ production in the absence and presence of PEI**


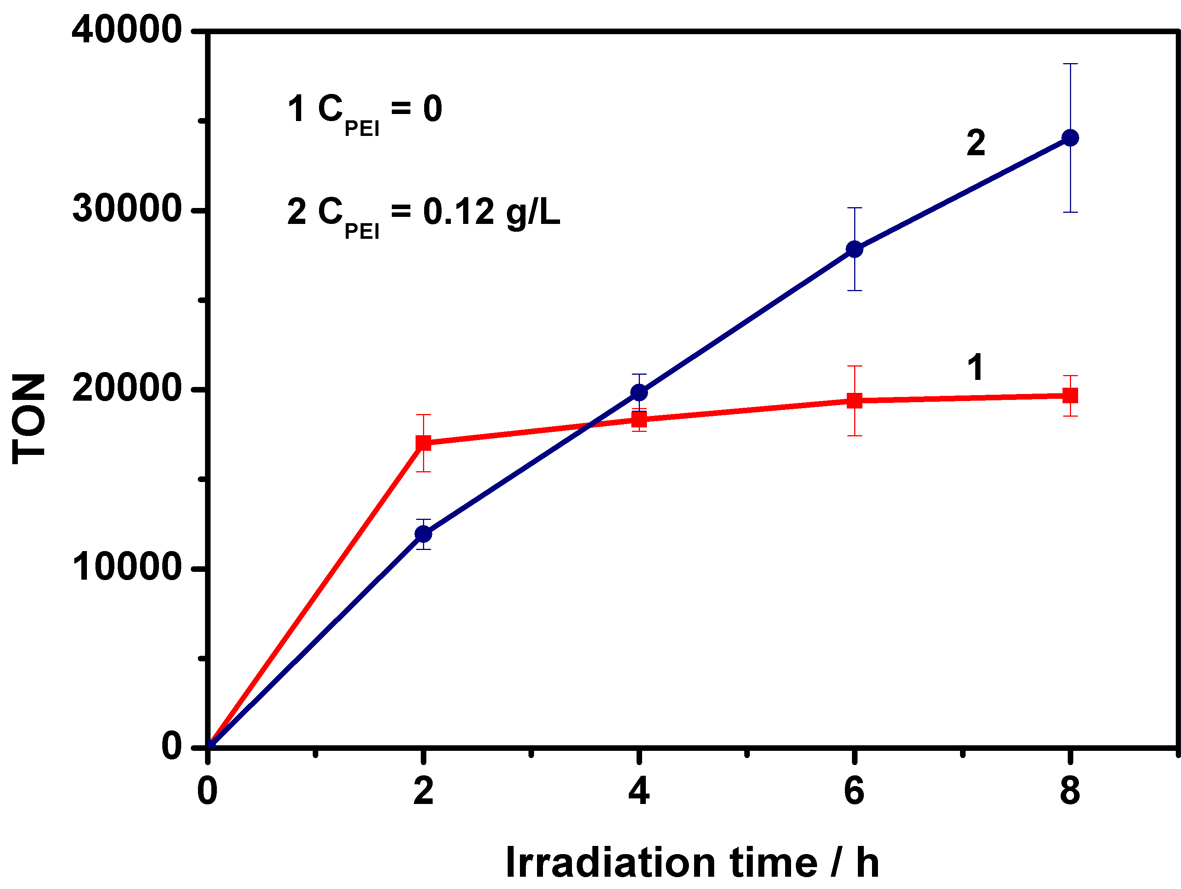


**Figure S3.** Photocatalytic H_2_ production in the absence and presence of PEI (0.12 g·L^-1^). Concentrations: CdSe QDs (5.8 × 10^-6^ mol·L^-1^), H_2_A (0.1 mol·L^-1^), PAA-*g*-Fe_2_S_2_ (0.25 g·L^-1^), pH 4.0-4.1.

**11. H_2_ production with different amounts of PEI**


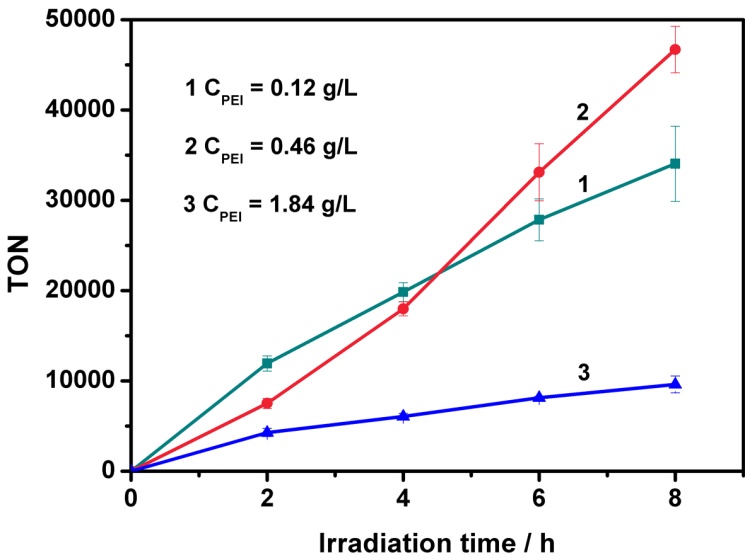


**Figure S4.** Photocatalytic H_2_ production with different amounts of PEI in the multi-component solution (0.12 g·L^-1^, 0.46 g·L^-1^ and 1.84 g·L^-1^). Concentrations: PAA-*g*-Fe_2_S_2_ (0.25 g·L^-1^), CdSe QDs (5.8 × 10^-6^ mol·L^-1^), H_2_A (0.1 mol·L^-1^), pH 4.0-4.1.

**12. DLS results of CdSe QDs with different amounts of PEI**


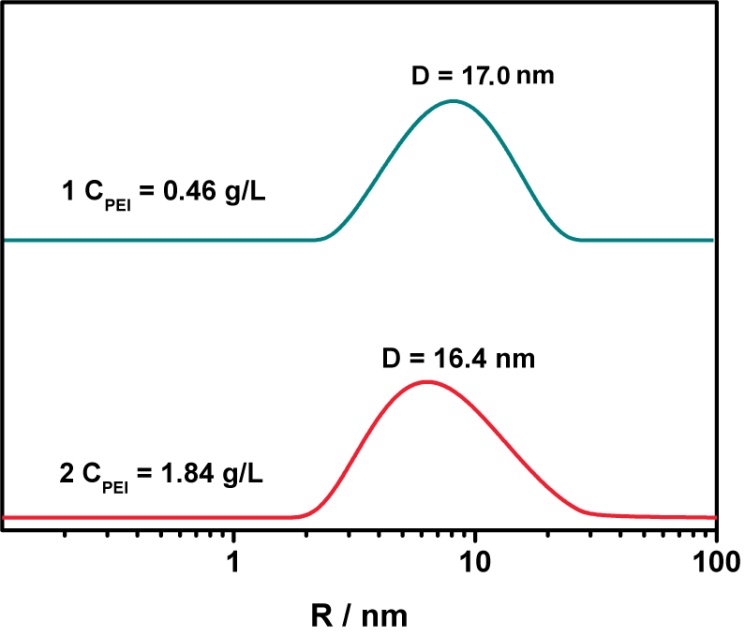


**Figure S5.** DLS results of CdSe QDs with different amounts of PEI in the multi-component solution (0.46 g·L^-1^ and 1.84 g·L^-1^). Concentrations: H_2_A (0.1 mol·L^-1^), PAA-*g*-Fe_2_S_2_ (0.25 g·L^-1^), CdSe QDs (5.8 × 10^-6^ mol·L^-1^), pH 4.0-4.1.

**13. H_2_ production with different amounts of H_2_A**


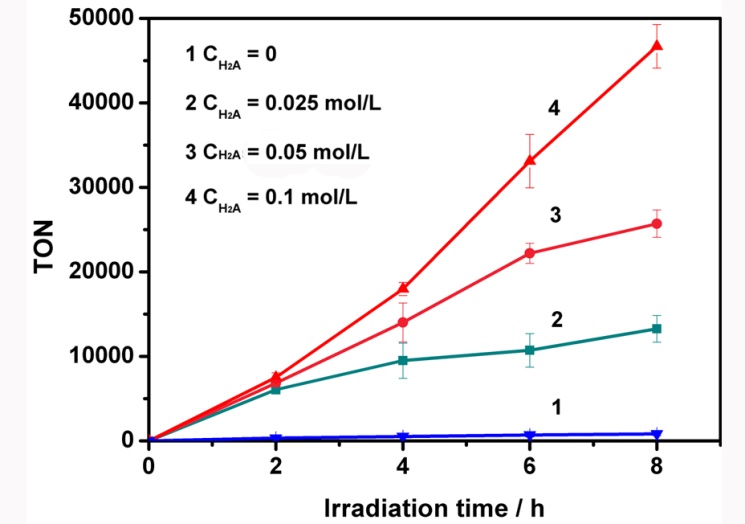


**Figure S6.** Photocatalytic H_2_ production with different amounts of H_2_A in the multi-component solution (0.1 mol·L^-1^, 0.05 mol·L^-1^, 0.025 mol·L^-1^ and 0 mol·L^-1^). Concentrations: PAA-*g*-Fe_2_S_2_ (0.25 g·L^-1^), CdSe QDs (5.8 × 10^-6^ mol·L^-1^), PEI (0.46 g·L^-1^), pH 4.0-4.1.

**14. H_2_ production in the absence and presence of Fe_2_S_2_ active site**


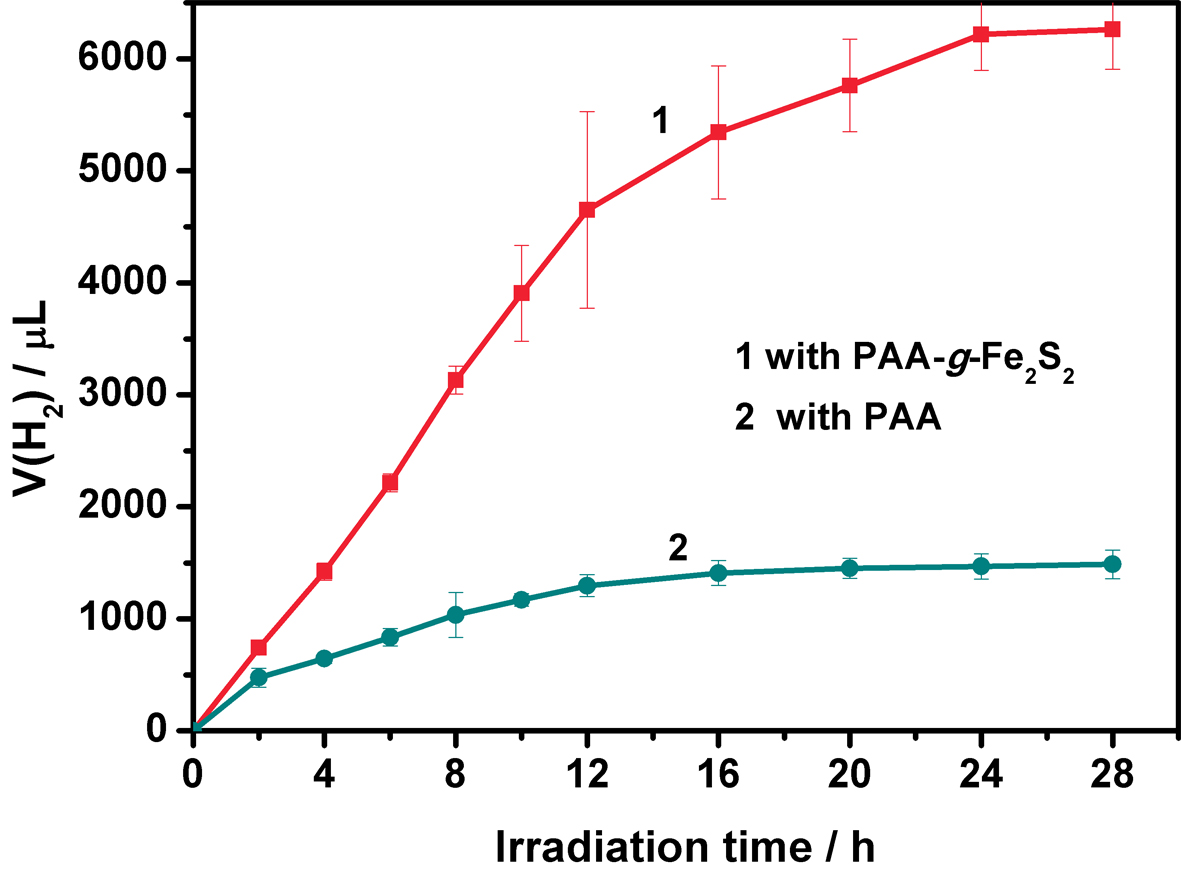


**Figure S7.** Photocatalytic H_2_ production in the absence and presence of Fe_2_S_2_ active site. Concentrations: CdSe QDs (5.8 × 10^-6^ mol·L^-1^), PAA-*g*-Fe_2_S_2_ (0.25 g·L^-1^), PAA (0.25 g·L^-1^), H_2_A (0.1 mol·L^-1^), PEI (0.46 g·L^-1^), pH 4.0-4.1.

**15. Emission spectra of CdSe QDs under different conditions**


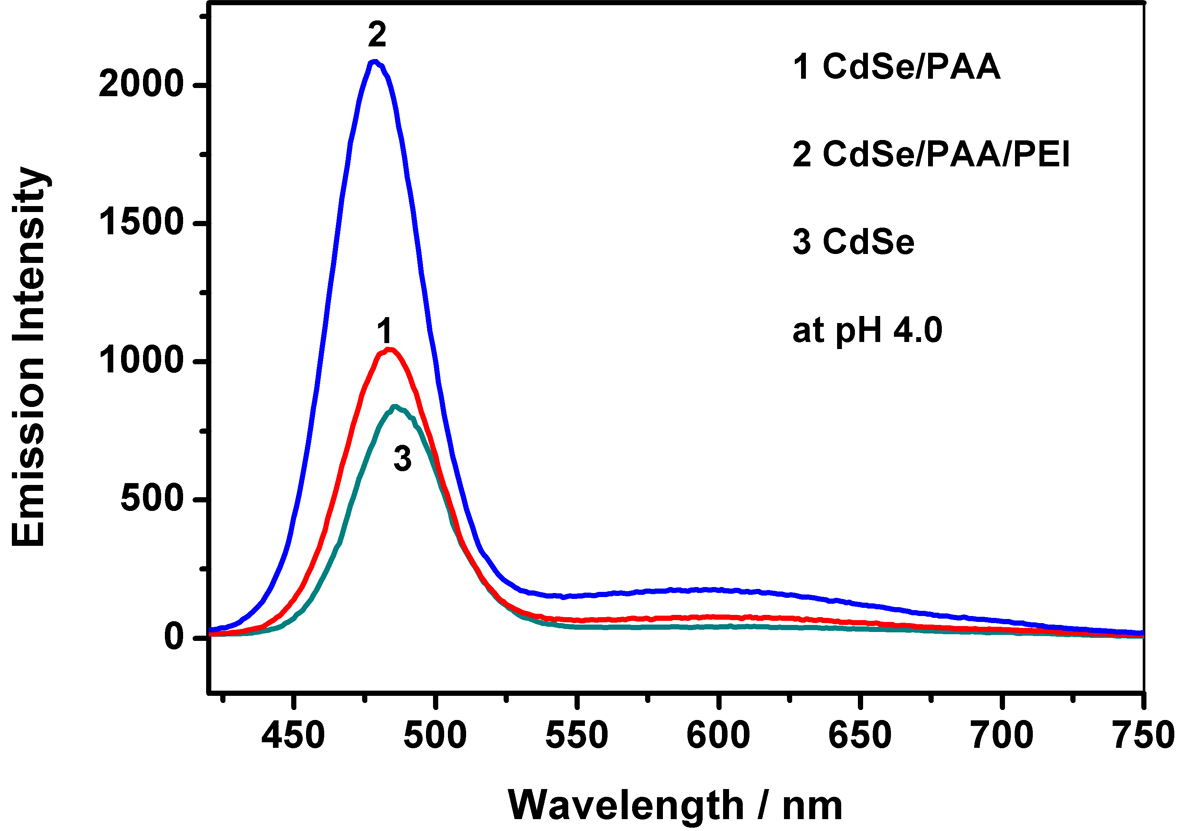


**Figure S8.** The emission spectra of CdSe QDs (1.6 × 10^-5^ mol·L^-1^) in the presence of PAA (line 1), in the co-presence of PAA and PEI (line 2) and in the absence of PAA and PEI (line 3) at pH 4.1.

**16. Photographs of PEI-modified CdSe QDs**


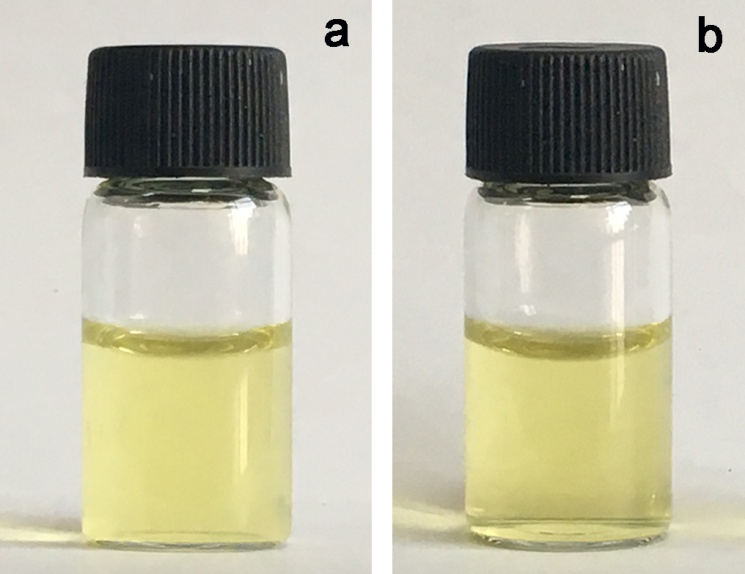


**Figure S9.** Photographs of the solution containing CdSe QDs (5.8 × 10^-6^ mol·L^-1^), PAA-*g*-Fe_2_S_2_ (0.25 g·L^-1^), H_2_A (0.1 mol·L^-1^), and PEI (0.46 g·L^-1^) at pH 4.0-4.1 before (a) and after (b) placed for 7 days.

**17. Emission decays of CdSe QDs with and without PEI**


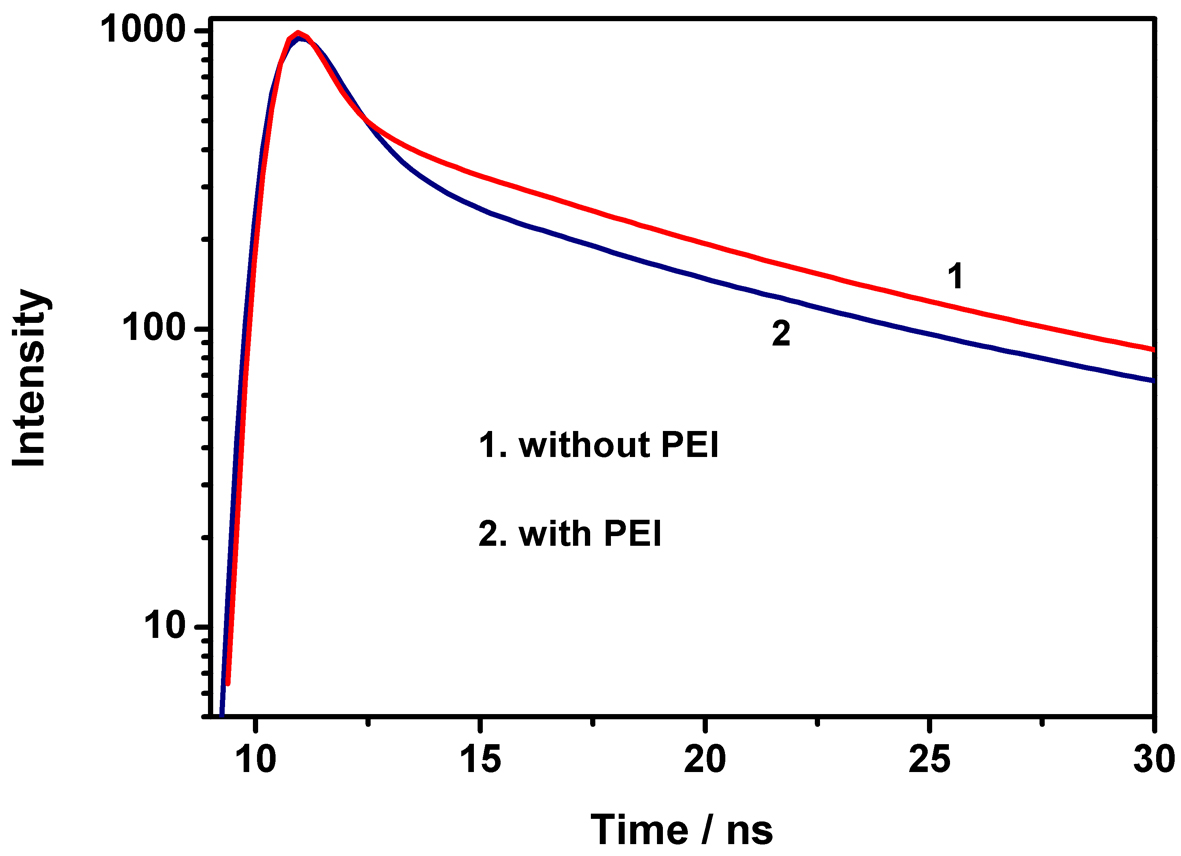


**Figure S10.** The emission decays of CdSe QDs (1.6 × 10^-5^ mol·L^-1^) in the presence of PAA (0.25 g·L^-1^) and in the co-presence of PAA (0.25 g·L^-1^) and PEI (0.46 g·L^-1^).

**18. Spectroelectrochemical absorption spectra of Fe^I^Fe^0^ species**


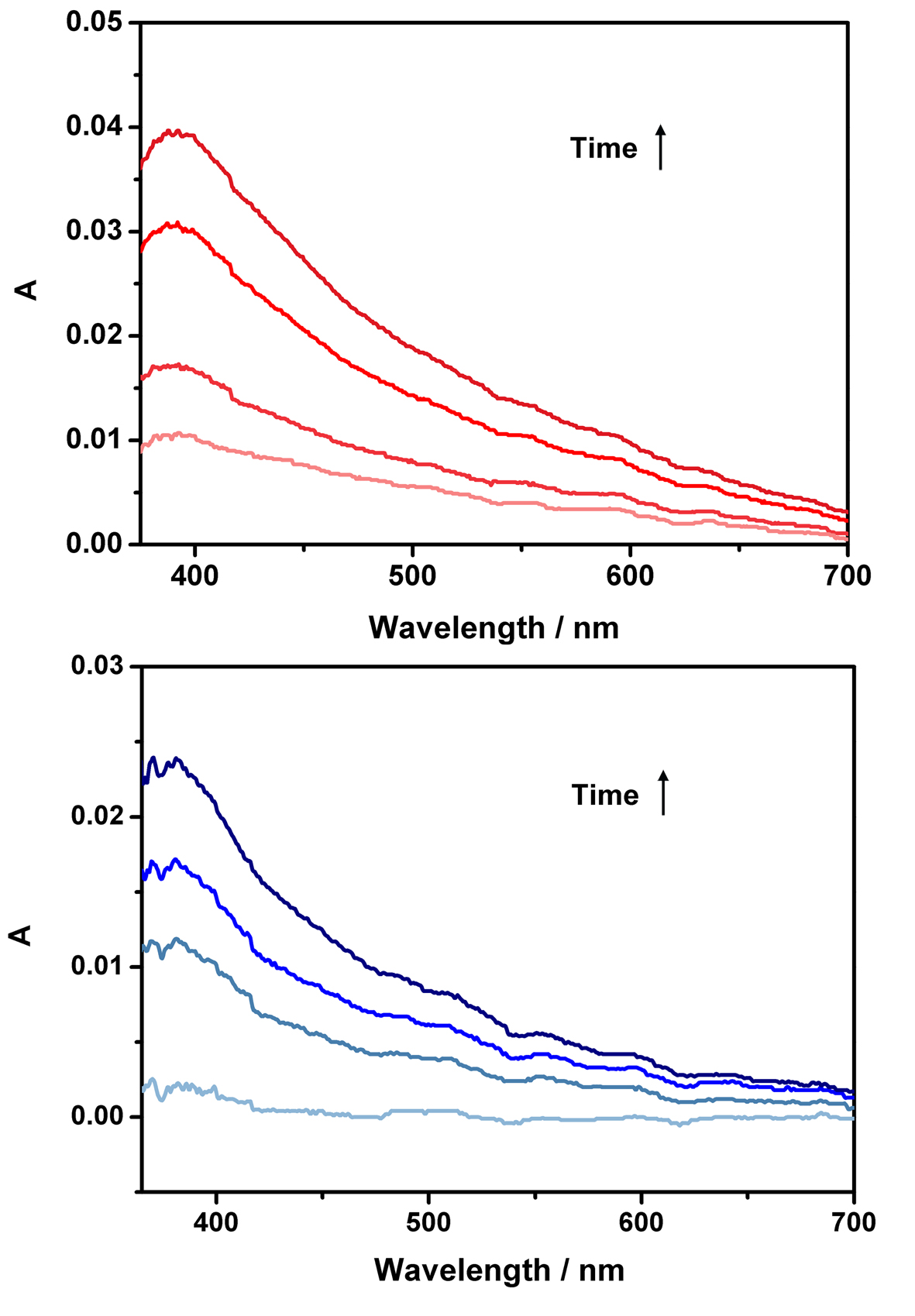


**Figure S11****.** Spectroelectrochemical absorption spectra of Fe^I^Fe^0^ species by reduction of PAA-*g*-Fe_2_S_2_ (0.25 g·L^-1^) in water in the absence (a) and presence (b) of PEI under constant voltage (-1.1 V *vs* Ag/AgCl). Working electrode: glass carbon; reference electrode: Ag/AgCl electrode; counter electrode: Pt wire; electrolyte: Na_2_SO_4_ (0.025 mol·L^-1^). All measurements were carried out under argon atmosphere.

**19. Spectroelectrochemical reduction of PAA and PEI**


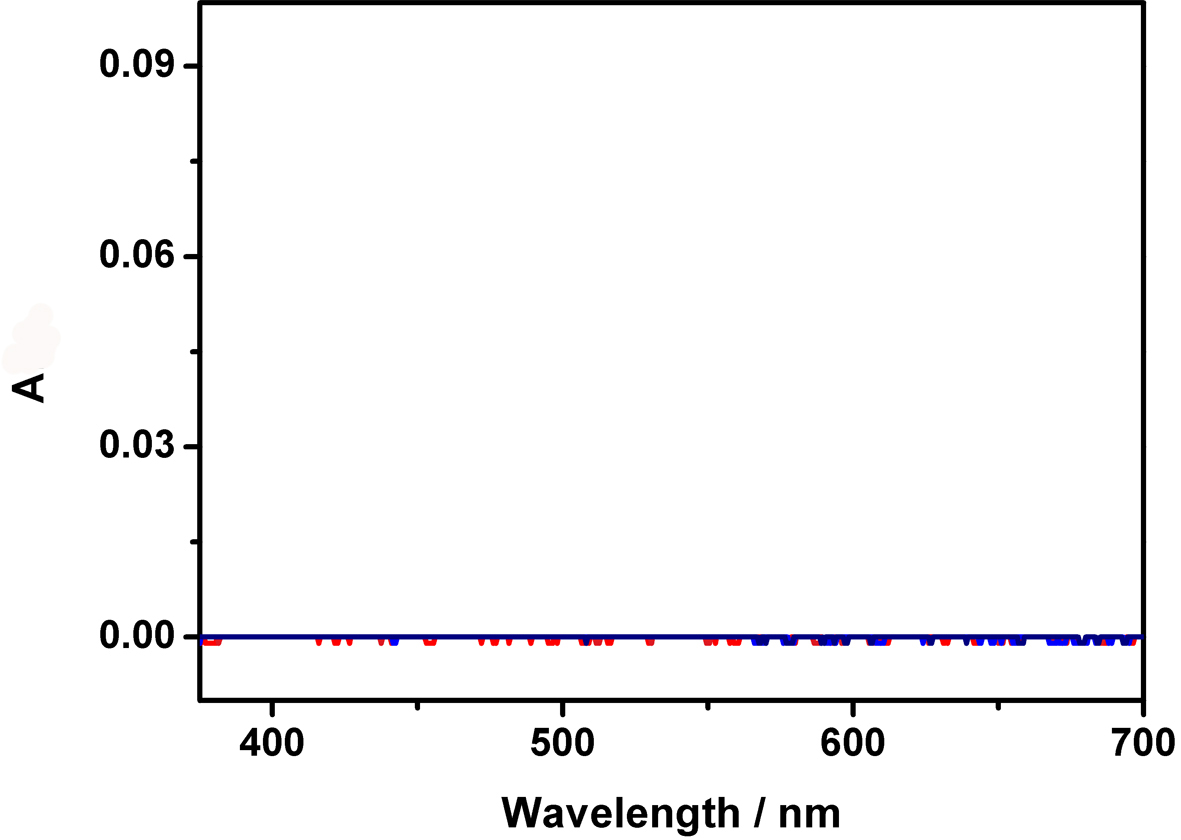


**Figure S12.** Spectroelectrochemical absorption spectra by reduction of PAA (0.25 g·L^-1^) in water in the absence and presence of PEI under constant voltage (-1.1 V *vs* Ag/AgCl). Working electrode: glass carbon; reference electrode: Ag/AgCl electrode; counter electrode: Pt wire; electrolyte: Na_2_SO_4_ (0.025 mol·L^-1^). All measurements were carried out under argon atmosphere.

**20. Stern-Volmer** **plot describing emission quenching of CdSe QDs**


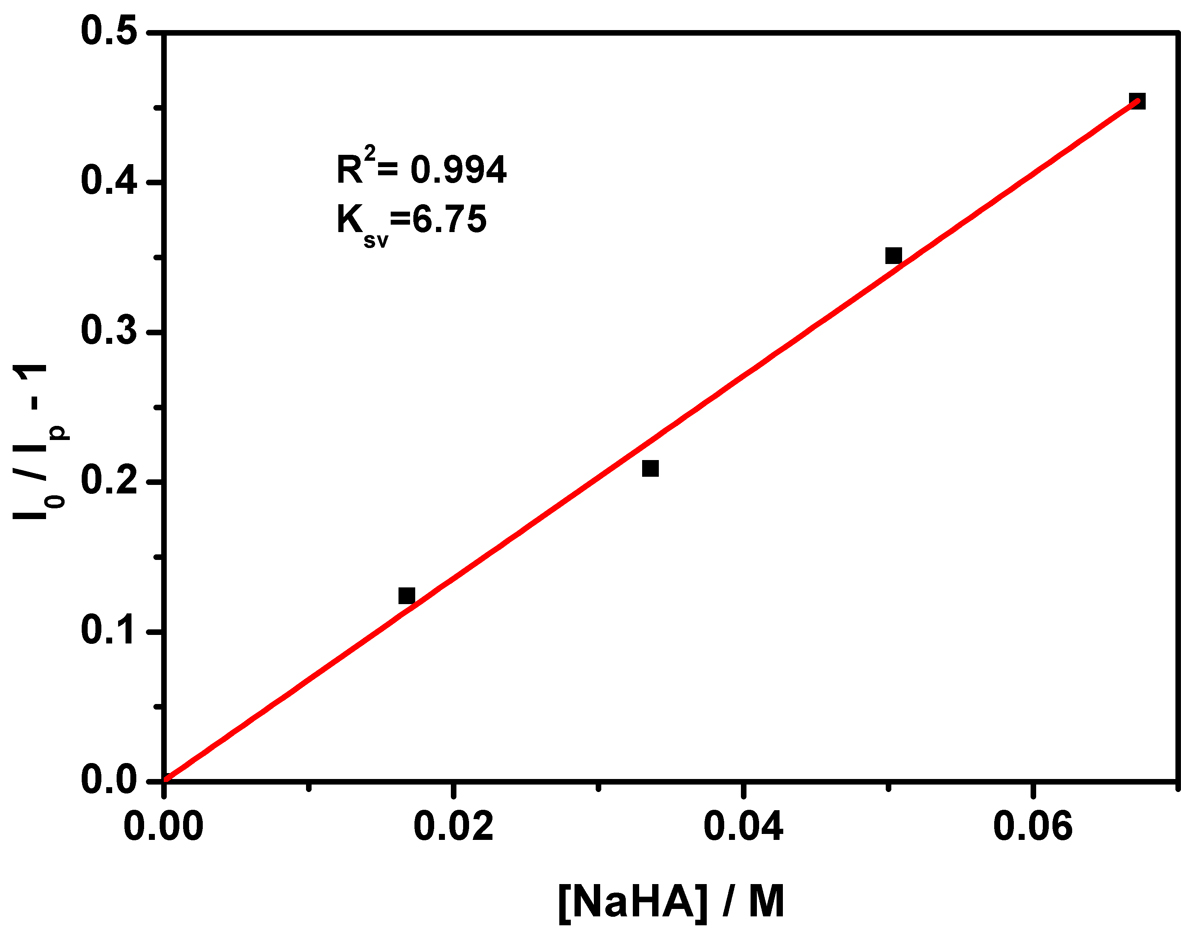


**Figure S13.** The emission quenching of CdSe QDs (1.6 × 10^-5^ mol·L^-1^) with increasing amounts of NaHA in the presence of PAA (0.25 g·L^-1^) by using Stern-Volmer equation.

**21.** **Stern-Volmer plot describing emission quenching of CdSe QDs with PEI**


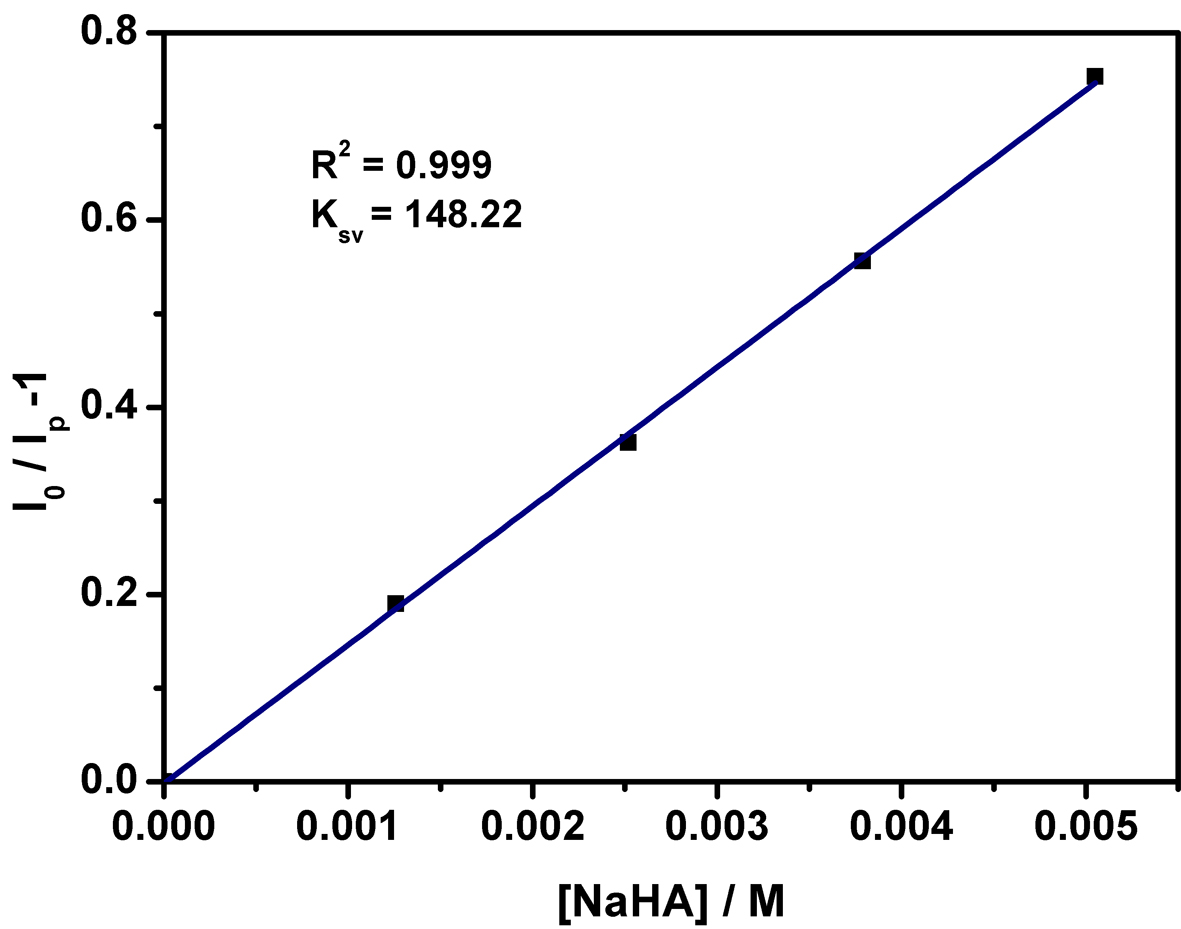


**Figure S14.** The emission quenching of CdSe QDs (1.6 × 10^-5^ mol·L^-1^) with increasing amounts of NaHA in the co-presence of PAA (0.25 g·L^-1^) and PEI (0.46 g·L^-1^) by using Stern-Volmer equation.

**22. Reference**

1. Yu, W.W., Qu, L., Guo, W. & Peng, X. Experimental Determination of the Extinction Coefficient of CdTe, CdSe, and CdS Nanocrystals. *Chem. Mater.* **15**, 2854-2860 (2003).
2. Sharma, S.N., Pillai, Z.S. & Kamat, P.V. Photoinduced Charge Transfer between CdSe Quantum Dots and p-Phenylenediamine. *J. Phy. Chem. B* **107**, 10088-10093 (2003).
3. Higgins, C. et al. Energy transfer in colloidal CdTe quantum dot nanoclusters. *Opt. Express* **18**, 24486-24494 (2010).
4. Baker, D.R. & Kamat, P.V. Tuning the Emission of CdSe Quantum Dots by Controlled Trap Enhancement. *Langmuir* **26**, 11272-11276 (2010).
5. Farrow, B. & Kamat, P.V. CdSe Quantum Dot Sensitized Solar Cells. Shuttling Electrons Through Stacked Carbon Nanocups. *J. Am. Chem. Soc.* **131**, 11124-11131 (2009).
6. Han, Z., McNamara, W.R., Eum, M.-S., Holland, P.L. & Eisenberg, R. A Nickel Thiolate Catalyst for the Long-Lived Photocatalytic Production of Hydrogen in a Noble-Metal-Free System. *Angew. Chem. Int. Ed.* **51**, 1667-1670 (2012).
7. Tseng, H.-W., Wilker, M.B., Damrauer, N.H. & Dukovic, G. Charge Transfer Dynamics between Photoexcited CdS Nanorods and Mononuclear Ru Water-Oxidation Catalysts. *J. Am. Chem. Soc.* **135**, 3383-3386 (2013).
8. Somasundaran, P., Healy, T.W. & Fuerstenau, D.W. Surfactant Adsorption at the Solid-Liquid Interface-Dependence of Mechanism on Chain Length. *J. Phys. Chem.* **68**, 3562-3566 (1964).
